# Evidence-based recommendations for application of construct-based splicing data in clinical variant classification

**DOI:** 10.1186/s13073-026-01746-3

**Published:** 2026-08-13

**Authors:** Daffodil M. Canson, George A. R. Wiggins, Eladio A. Velasco-Sampedro, John F. Pearson, Logan C. Walker, Amanda B. Spurdle

**Affiliations:** 1 Population Health Program, QIMR Berghofer, Herston, QLD 4006 Australia; 2 Department of Pathology and Molecular Medicine, University of Otago, Christchurch, New Zealand; 3 Splicing and Genetic Susceptibility to Cancer, Instituto de Biomedicina y Genética Molecular de Valladolid (IBGM), Consejo Superior de Investigaciones Científicas - Universidad de Valladolid (CSIC-UVa), Valladolid, 47003 Spain; 4 Department of Medicine, University of Otago, Christchurch, New Zealand

**Keywords:** RNA splicing, Variant interpretation, Construct-based assays, Massively parallel splicing assays (MPSA), Minigenes, SpliceAI, SAI‑10k‑calc, Clinical calibration, ACMG/AMP guidelines, Loss‑of‑function (LOF)

## Abstract

**Background:**

Minigene RT-PCR assays are widely used to assess variant impact on splicing, with increasing reports of massively parallel splicing assays (MPSAs) demonstrating potential to upscale diagnostic use of construct-based data. This study conducted a comprehensive evaluation of > 41,000 variants from construct-based splicing assays, to build evidence-based recommendations to support the consistent application of such assays in clinical variant interpretation.

**Methods:**

Seven MPSAs were reviewed for design limitations, and their discriminatory performance evaluated by assessing: assay score distribution; consistency of splice-impact thresholds with expectations based on SpliceAI predictions. A traditional minigene RT-PCR dataset comprising 673 variants from 14 studies was analysed to: assess potential for SpliceAI score to predict level of aberration; demonstrate performance of SpliceAI-10k calculator (SAI-10k-calc) to accurately predict variant-induced splicing events; calibrate evidence strength towards or against spliceogenicity based on SpliceAI score. Traditional minigene results were compared to patient-derived RNA results, and calibrated for evidence strength towards or against pathogenicity using assertions from ClinVar.

**Results:**

MPSA datasets lacked specific information on variant-induced transcripts and had design limitations preventing detection of some aberrant splice events. Assay scores generated by five of seven MPSAs were unable to differentiate aberrant from natural splicing events. Traditional minigene results showed high predictive agreement between predicted and observed variant-induced events: SpliceAI score of 0.285 showed 90% sensitivity and 90% specificity for separating no/low versus intermediate-to-complete aberration, and agreement was 87% for aberration type using SAI-10k-calc. Minigene and patient-derived RNA results showed 89% (81/91) complete or high concordance for predominant transcripts. Clinical calibration of minigene results showed that high (≥ 80%) or low (≤ 20%) expression of variant-induced loss of function (LOF) transcripts provide strong evidence towards or against pathogenicity, respectively. Evidence-based recommendations for design and critique of construct-based assays were built based on these and other findings.

**Conclusions:**

MPSA screens require review of design limitations and performance evaluation before considering their suitability for clinical variant interpretation. Well-designed multi-exon minigene assays can provide quantitative RNA results to supplement patient-derived RNA findings, and clinical calibration justifies their use in the diagnostic setting. Altogether, these findings support more consistent application of RNA evidence in clinical practice.

**Supplementary Information:**

The online version contains supplementary material available at https://doi.org/10.1186/s13073-026-01746-3.

## Background

Identifying germline pathogenic variants is important for directing clinical care of patients and their families. In 2015, the American College of Medical Genetics and Genomics (ACMG) and the Association for Molecular Pathology (AMP) published a framework for classifying variants using multiple categories and degrees of evidence [1]. Further, the Clinical Genome Resource (ClinGen) consortium [2] has developed more detailed recommendations, termed Criteria Specifications, for application of different evidence types to promote more consistent clinical variant curation using the above framework. However, interpreting the clinical significance of variants in disease susceptibility genes remains a significant diagnostic challenge. Furthermore, the body of genetic knowledge contributing to variant classification is notably biased according to ethnicity. For example, allele frequency data used to guide the assessment of genetic variants are not representative of minority population groups, contributing to inequities in genomic variant classification.

RNA diagnostics has emerged as an important strategy for establishing the clinical relevance of gene variants. Recent studies have shown that RNA sequencing (RNA-seq) improves the diagnostic yield by up to 25%, enabling more informed clinical management of patients [3–7]. Importantly, RNA diagnostics also helps mitigate the reliance on biased population-specific reference data, offering an orthogonal approach to DNA-based variant interpretation. The current ACMG/AMP framework [1], supplemented with splicing-specific recommendations [8], denotes five ACMG/AMP codes for capturing potential splicing impact based on variant location, including: (1) splice donor/acceptor ± 1,2 dinucleotide positions (PVS1); (2) similarity of predicted impact compared to a known (likely) pathogenic variant (PS1); (3) variants located at other positions (PP3, BP4); and (4) additional evidence against pathogenicity for synonymous or intronic variants with no predicted impact on splicing and thus no other likely mechanism (BP7). While bioinformatic tools that predict variant impact on splicing play a critical role as the initial step in variant assessment, RNA assay data adds value by: (1) confirming that a variant is spliceogenic i.e. alters native splicing profile compared to controls; (2) determining whether the impact on splicing is complete or partial; and (3) revealing the altered transcript profile, to infer protein-level impact e.g., spliceogenic predicted loss of function (LOF), functional or uncertain function [9].

Assay-based detection of splicing using RT-PCR and RNA-seq of RNA from patient samples has been used to measure impact on splicing associated with the variant allele, ranging from complete (i.e., no reference transcript), partial (i.e., varying levels of reference transcript), to none [10–12]. However, results can vary based on factors such as tissue source, transcript isoform complexity, allele expression bias, and assay sensitivity [13], and it is difficult to track a partial effect for intronic variants using patient RNA. These limitations have led to the application of allele-specific minigene models in the research and diagnostic setting, not only to quantify allele-specific variant effects on splicing, but to enable variant impact assessment where patient RNA is unavailable [14–16]. More recently, massively parallel splicing assays (MPSAs) have enabled high-throughput functional assessment of thousands of variants in a single experiment [17–23]. These studies generate large datasets capturing quantitative effects of sequence variation on splicing and potentially provide a rich resource for benchmarking computational prediction tools and informing clinical variant interpretation.

The existing ClinGen recommendations for use of splicing prediction and assay data provide high-level considerations for application of construct data for variant classification, and suggest “a conservative approach would be to apply information from construct data alone at lower weight in the absence of calibration of an experimental system against clinical data for proven spliceogenic and non-spliceogenic variants” [8]. Despite the increased availability of splicing data from construct-based assays, and their potential to provide valuable insights into the type and level of impact induced by sequence variants, for MPSA data especially there has been limited evaluation of splicing data consistency across assay platforms, cell models, and the pipelines used to process RNA data. In this study, we conducted a comprehensive analysis of the spliceogenic effects of approximately 41,000 variants, in 5,458 genes from 21 published construct-based datasets that utilised an MPSA method or traditional minigene RT-PCR assays. We then used findings to develop recommendations about the utility of such data for diagnostic variant classification following the ACMG/AMP framework.

## Methods

### Study datasets

RNA splicing data used in this study were accessed from 21 published construct-based datasets − 7 MPSAs [17, 19–24] and 14 traditional minigene RT-PCR assays (hereafter called traditional minigene assays) [25–38]. Overall, the MPSA dataset included 41,128 variants, while the traditional minigene dataset included 673 variants. Details of all datasets are provided in Additional file 1: Table S1.

The MPSA datasets selected for analysis met the following inclusion criteria: (1) used a construct-based assay approach to assess the impact of sequence variants on RNA splicing; and (2) analysed more than 100 variants.

To limit potential variability due to high-level differences in assay design and execution (such as use of single- versus multi-exon minigenes, inclusion of sufficient intronic flanking sequence, assays in replicate, use of experimental controls), the traditional minigene assay datasets were intentionally selected from a single laboratory (author E.A.V.-S.). These datasets were generated using multi-exon minigene constructs to assess gene variants in established cancer susceptibility genes and applied the same method to semi-quantify the transcript products: (1) transfected cells were treated with cycloheximide, an inhibitor of nonsense mediated decay (NMD); and (2) fluorescent RT-PCR products were assessed by capillary electrophoresis on an automated DNA sequencer.

### MPSA data preparation

Data from each study was downloaded from either the manuscript, supplementary information or an online repository (Additional file 1: Table S1). A step-wise and study-specific approach was taken to identify the relevant transcript for SpliceAI prediction. O’Neill et al. [19] and Rong et al. [22] provided the relevant Ensembl or RefSeq transcript identifiers. For Adamson et al. [17] and Chong et al. [18], transcripts were identified based on matching the provided exon coordinates with MANE and RefSeq transcripts exon coordinates. Patel et al. [20], Soemedi et al. [23] and Rhine et al. [21] provided no exon coordinates for the respective assay designs, therefore transcripts were selected based on the variant coordinate, prioritising the MANE transcripts. Additionally, for Soemedi et al. [23], we assumed alleles were provided for the RNA transcript and not DNA. As SpliceAI requires variants to be annotated based on the DNA sequence, the allele information provided by Soemedi et al. [23] was compared to the DNA reference at the given locus; for variants discordant to the DNA reference (*n* = 2,483), the complement of reported reference and variant allele was used. See Additional file 1: Table S2 for detailed summary of MPSA designs, characteristics and study-reported thresholds, including limitations to detect certain types of aberrations.

### Evaluation of MPSA data for clinical application

We employed a multi-step approach to evaluate the discriminatory performance of MPSAs and establish their potential clinical utility.

1. To investigate whether each MPSA effectively differentiated spliceogenic from non-spliceogenic variants using study-reported thresholds, the distribution of assay scores (e.g. delta percentage spliced in (PSI)) were visually assessed using a histogram; the presence of a bimodal or multi-modal distribution was considered as evidence for the ability of the assay to demarcate spliceogenic and non-spliceogenic variants. Unimodality was tested using Hartigan’s dip test with the dip.test package in R [39].
2. We assessed if the study-reported thresholds for detecting spliceogenic variants for different MPSA datasets were consistent with expectations based on SpliceAI predictions, using max delta score (hereafter referred to as SpliceAI score) thresholds from previous calibration of this tool for use in ACMG/AMP variant classification [8]. Splicing impact was only calculated for the transcript used in the relevant construct (see section ‘MPSA data preparation’).
3. To determine if spliceogenic variants with predicted high impact were enriched within the splice site motifs as expected, particularly at the splice donor/acceptor ± 1,2 dinucleotide positions, we visually examined the assay score distribution based on variant location. To further examine the capacity of MPSAs to detect variants highly likely to be spliceogenic, we selected the subset of variants located at the splice donor/acceptor ± 1,2 dinucleotide positions, and plotted their assay and SpliceAI scores to determine if the MPSA consistently identified variants predicted to have impact as spliceogenic.

### Data formatting and standardization for traditional minigene studies

Minigene splicing data for 673 variants in seven cancer susceptibility genes (*ATM*,* BRCA2*,* CHEK2*,* PALB2*,* RAD51C*,* RAD51D*, and *TP53*) were extracted from 14 publications (Additional file 1: Table S1), and formatted for consistent presentation of semi-quantitative RT-PCR results (Additional file 1: Table S3). Assay results indicated the proportions of specific transcript products and the corresponding functional consequence in terms of effect on reading frame and the introduction of premature termination codon (PTC). To determine the level of splicing aberration induced by a variant relative to the level produced by the wild type (WT) construct, the reduction of canonical transcript (CT) was computed using this formula: [(CT_WT_ – CT_variant_)/CT_WT_] x 100. A 100% CT reduction indicated complete splicing aberration (that is, no canonical transcript produced by the variant allele). The variants were binned into four levels of splicing aberration based on CT reduction: high/complete (≥ 80% to 100%), intermediate (> 20% to < 80%), low (> 0% to ≤ 20%), or no aberration (≤ 0%). The no aberration category included variants that increased the level of canonical transcript (that is, negative measure of CT reduction).

### Performance evaluation of splicing prediction tools using the traditional minigene dataset

Prediction tool evaluation was designed to build on our previous work, where SpliceAI was selected as the best-performing tool, and used to exemplify tool calibration providing evidence against/towards spliceogenicity [8]. Performance evaluation covered two aspects critical for the use of prediction data in variant curation. First we assessed if SpliceAI [40] score could be used to predict different levels of aberration: no vs. low-to-complete, no/low vs. intermediate-to-complete, and no-to-intermediate vs. high/complete. The maximum raw SpliceAI score was defined as the maximum probability of altered splicing across the four output probabilities at a maximum distance of 10,000 nucleotides (± 4,999 nucleotides from the variant of interest). To determine the optimal thresholds for separating the aberration levels, we performed receiver operating characteristic (ROC) analysis using the pROC R package [41] and obtained the Youden index, which helped identify the optimal binary cut-off point on the ROC curve where the test performs best, balancing sensitivity and specificity. To determine the magnitude and direction of the correlation between the SpliceAI prediction and level of splicing aberration, we computed Kendall’s tau.

We then examined the ability of the SpliceAI-10k calculator (SAI-10k-calc) [42], recently modified to incorporate SpliceAI alternate scores [43], to accurately predict the variant-induced splicing events. For this assessment, variants with intermediate to complete aberration (> 20% CT reduction) were used as the positive control set, and no/low aberration (≤ 20% CT reduction) as the negative control set, as previously described [43]. The concordance of SAI-10k-calc transcript predictions with the minigene assay results were calculated based on the following criteria: concordance of positive predictions (one or more predicted events were detected at > 20% of total transcript pool), concordance of negative predictions (agreement between predicted no aberrant transcript and no/low aberration detected in the assay), and overall concordance (total concordant positive and negative predictions). The SAI-10k-calc algorithm for predicting exon skipping and intron retention requires the loss of both native acceptor and donor splice sites. In instances where at least one of the SpliceAI-predicted losses affects a cryptic splice site, SAI-10k-calc flags the variant with an annotation “lost site/s do not match consensus”; in this study, we modified the flag into “lost site/s do not match native site” for clarity. For variants flagged by SAI-10k-calc with “lost site/s do not match native site” under the “Exon_skipping_aaseq” or “Intron_retention_aaseq” fields, we manually inspected the SpliceAI scores using SpliceAI-visual [44] based on the decision flowchart in Additional file 2: Fig. S1 to obtain their final transcript prediction. For variants where the predicted aberrant transcript was absent or expressed at low level and other dominant transcript/s were detected in the assay, we additionally checked if the predicted and observed transcripts had the same functional consequence (PTC or in-frame), and considered them as concordant in a separate comparison.

We also assessed whether knowledge of naturally occurring splicing events improved the performance of SAI-10k-calc to predict splicing events, by considering information captured in the SpliceVault resource [45], a database of common splicing events observed in reference RNA-sequencing samples. To obtain the SpliceVault 300 K-RNA Top-4 alternative splicing events [45], we used the Ensembl Variant Effect Predictor (VEP) v113.4 [46] with the SpliceVault VEP plugin (https://github.com/Ensembl/VEP_plugins/blob/release/113/SpliceVault.pm), which annotates variants with SpliceAI-predicted loss of native splice sites only. We chose the Ensembl transcript identifier equivalent to the RefSeq MANE Select transcript. SpliceVault results were considered as concordant if the Top-4 events matched with at least one aberrant transcript exhibiting > 20% expression in the minigene assay. Since native splice site loss is mostly caused by splice site motif disruption, SpliceVault concordance calculations were limited to variants in the splice donor and acceptor motifs, defined as in Walker et al. [8] (donor motif - the last three nucleotides of the exon to six nucleotides downstream of the exon; acceptor motif - the first nucleotide of the exon up to 20 nucleotides upstream of the exon).

### Calibration of SpliceAI for computational code application, using quantitative RNA results

The likelihood ratio (LR) of spliceogenicity for variants located outside the splice donor/acceptor ± 1,2 dinucleotide positions was estimated using the formula previously reported [47]. We initially used the previously established SpliceAI score thresholds [8] based on analysis of an RNA splicing data truth set that did not capture quantification of transcript levels (that is, categorization of splicing aberration was binary, recorded simply as Yes or No). Quantitative splicing data from the traditional minigene dataset analyzed in this study enabled an assessment of SpliceAI thresholds for optimal prediction of no aberration versus aberration at a given minimum level, using the Youden index to estimate the optimal thresholds [48] Thresholds were estimated for best separation of no aberration versus low-to-complete, and for no/low aberration versus intermediate-to-complete aberration. We then used these thresholds to estimate LRs towards spliceogenicity (at the denoted minimum levels), and equated each LR with an evidence strength category according to the Bayesian framework of the ACMG/AMP variant classification guidelines [49].

### Evaluation of traditional minigene RNA results for diagnostic variant classification

#### Comparison of minigene and patient-derived RNA results

Traditional minigene assay results were compared with previously published splicing data derived from patient RNA. For positive patient RNA assay outcomes, concordance was assessed between the traditional minigene assay dataset and reported patient findings for all seven genes. Concordant results were defined as either complete concordance of all detected transcripts or high concordance where a single patient-derived transcript corresponded to the most abundant transcript observed in the minigene assay, or vice versa. Discordant results were defined as complete disagreement, where no transcripts overlapped between the two systems, or low concordance where patient transcript(s) aligned only with minor transcripts from the minigene assay. For variants with multiple patient RNA findings, outcomes were prioritised in the following order when assigning concordance: complete, high, low, and no concordance. For example, if a variant had two patient findings, one a complete concordance and the other a high concordance involving the most abundant transcript, it was classified as a complete concordance. Negative patient RNA assay outcomes were evaluated only for *BRCA2*, leveraging our previously published in-house resource of splicing data extracted from publications, that provides comprehensive splicing information for this gene [8].

#### Clinical calibration to derive strength of evidence applicable to construct-based RNA findings

All variant annotations relevant for clinical calibration are provided in Additional file 1: Table S4. Classifications were extracted from ClinVar (October 2025), and crossmatched to the variants in the traditional minigene assay dataset. A total of 513 variants were reported at least once in ClinVar, with summary classification as follows: 12 Benign (B); 10 Benign/Likely benign (B/LB); 43 Likely benign (LB); 115 Uncertain significance (VUS); 111 conflicting classifications; 41 Likely pathogenic (LP); 81 Pathogenic/Likely pathogenic (P/LP); 100 Pathogenic (P). For variants with conflicting classifications of pathogenicity, application of a simple majority rule resolved summary classification for 109 variants: 13 B/LB, 6 LB, 73 VUS, 9 LP, 8 P/LP.

As a critical step to perform a calibration that correlates the construct-based findings with clinical evidence of variant pathogenicity (and not simply variant spliceogenicity), RNA findings were reviewed to determine the percentage of transcripts predicted to cause gene LOF (RNA transcripts predicted non-coding, predicted protein truncating leading to NMD, predicted protein truncating without NMD but impacting a critical domain, and/or predicted to encode proteins lacking critical structural/functional motifs [9]. A transcript was assigned as predicted LOF if annotated as a PTC transcript, or if in-frame and eligible to be assigned at least moderate weight as reported in the original minigene RT-PCR publications (Additional file 1: Table S1), or for *BRCA2*, following the ClinGen ENIGMA Variant Curation Expert Panel (VCEP) specifications V1.2 [50]. The summed percentage of all predicted LOF transcripts observed for each variant was categorised as follows: ≤20%; >20% and < 50%; ≥50% and < 80%; ≥80%.

Variants were assigned a variant type using VEP consequence annotation, to allow consideration of an alternative mechanism of pathogenicity for variants annotated as nonsense, frameshift or missense variants at the DNA level; stratification of splice region variants according to location inside or outside the highly conserved splice site dinucleotide positions. Variants were excluded from the calibration reference sets if they met the following criteria: summary classification as VUS (*n* = 188) or conflicting (*n* = 2); summary classification P/LP and DNA level consequence as nonsense, frameshift or deletion within a critical domain (*n* = 43), of which only 5 displayed a high level of splice impact; or summary classification P/LP and missense consequence (*n* = 28), of which only 7 displayed high level of splice impact.

Variants considered to be potential outliers for calibration were then reviewed for pathogenicity. This resulted in the exclusion of two variants with a (likely) pathogenic assertion and ≤ 20% predicted LOF transcripts. *BRCA2* c.9501G > A (p.Glu3167=), with a single submitter, no assertion criteria, no star; classification VUS following the ClinGen ENIGMA BRCA1/2 VCEP specifications V1.2 [50], meeting PM2_Supporting (1 point), and no bioinformatic code (SpliceAI score 0.16). *PALB2* c.108 + 2T > C, LP with a single submitter and no RNA data; this variant is designated as no PVS1 code applicable following the ClinGen Hereditary Breast Ovarian Pancreatic VCEP specifications V1.2 [51]. Classification remained unchanged for another five potential outlier variants assessed by traditional minigene assays: two B/LB classification and ≥ 50% predicted LOF transcript assays (*ATM* c.1066-6T > G; *BRCA2* c.441 A > G p.Gln147=); two P/LP *PALB2* variants with complete impact on splicing, but with < 50% predicted LOF transcripts due to exclusion of a transcript reaching only PVS1_Supporting (*PALB2* c.211 + 1G > A and c.211 + 2T > C); and similarly the *BRCA2* c.9257-1G > C variant with multiple P submissions (some supported by internal laboratory data), and complete impact on splicing resulting in an in-frame transcript reaching only PVS1_Supporting level. Additional review of ClinVar entries was then performed for all reference set variants located outside of the splice donor/acceptor ± 1,2 dinucleotide positions, to assess potential for circularity due to use of the minigene data as the only RNA source for an individual variant classification (Additional file 1: Table S5). An additional variant, *BRCA2* c.7875 A > G (p.Arg2625=), was excluded since the two submitters that did not use the minigene data had conflicting assertions of VUS or LB.The final reference sets for clinical calibration were comprised of 83 B/LB variants (all outside the splice donor/acceptor ± 1,2 dinucleotide positions), and 166 P/LP variants (28 outside the splice donor/acceptor ± 1,2 dinucleotide positions).

LR estimates towards or against pathogenicity were estimated by comparing the proportion of variants meeting different splice percentage categories within the benign versus pathogenic reference sets, using the statistical method detailed previously [47]. The LR estimates were then used to assign weights for or against pathogenicity following recommendations arising from Bayesian modelling of the ACMG/AMP guidelines [49].

## Results

### Characterisation of MPSA study designs

**Fig. 1:**
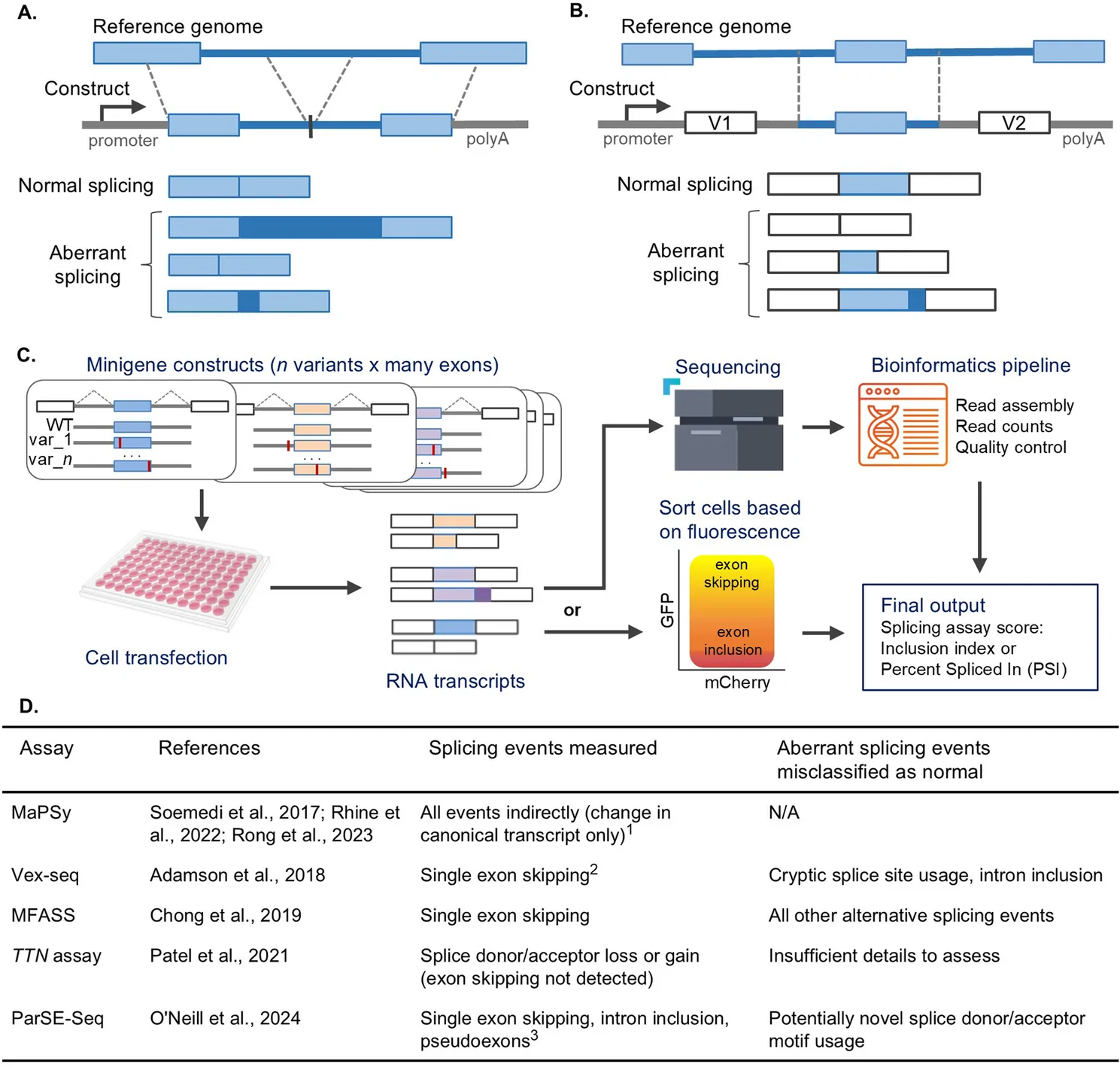
MPSA minigene construct design and assay workflow. **A** An exon-intron-exon construct design including only a portion of exonic and intronic sequences surrounding the donor and acceptor splice site pair as test sequence. Deep intronic sequences are deleted when necessary to fit the inserted test sequence within the 500-bp size limit. **B** A chimeric three-exon construct design containing a single test exon with flanking intronic sequences (blue) inserted between two constant vector exons (V1 and V2). **C** In MPSA, numerous wild type (WT) and variant minigene constructs are transfected into cells, then the RNA splicing products are pooled and sequenced, and the final splicing outcomes are bioinformatically determined. Alternatively, exon skipping/inclusion are measured by cell sorting based on fluorescence. **D** Splicing products measured by MPSAs included in this study. ^1^Indirectly measures all events possible within the limitation of a single-exon minigene. ^2^Measured by counting the number of introns after alignment (based on code review). ^3^Unclear if pipeline handles previously unannotated splice junctions

To determine whether MPSA results can be used for clinical variant classification, we evaluated two types of splicing studies: (1) gene-specific assays that assessed the impact of variants in one or a few genes for which the clinical validity of gene-disease relationship is established [19, 20] and (2) broad MPSA screens including hundreds of genes that were selected irrespective of gene-disease mechanism [17, 18, 21–23]. The study by Patel and colleagues used a construct with a 500 bp exon-intron-exon test sequence including partial exonic and intronic sequences surrounding the splice region (Fig. 1A), and all remaining studies evaluated constructs with a single test exon inserted between constant vector exons (Fig. 1B). The gene-specific ParSE-seq assay [19] used minigene constructs with a similar design to traditional single test exon splicing assays such that a wide range of exon sizes of up to ~ 350 bp and 125–250 bp of flanking intronic sequences were included (Additional file 1: Table S2). The broad MPSA screens, including Massively Parallel Splicing Assay (MaPSy) [21–23], Variant exon sequencing (Vex-seq) [17], and Multiplexed Functional Assay of Splicing using Sort-seq (MFASS) [18], used minigene constructs with inserted sequences limited to ~ 170 bp. These broad screens included test exons of ≤ 120 bp in size and shorter flanking intronic sequences with a lower limit of 40–50 bp upstream and 15–30 bp downstream of the exon. To allow for the inclusion of variants in larger exons (120–500 bp), Rong and colleagues designed a ‘half exon’ construct that incorporated the 100 bp of context-specific sequence (including the nearest splice site) with a common exon sequence and the other splice site. Generally, the minigene constructs were transfected into cells, and the splicing outcomes were measured in a pooled manner with sequencing data as quantitative readout of the splicing impact of variants (Fig. 1C). For the MFASS method, splicing impact quantification was done by fluorescence-activated cell sorting based on green fluorescent protein and mCherry reporters [18] (Fig. 1C). Detailed characterisation of study designs is shown in Additional file 1: Table S2.

### MPSA performance evaluation

All seven published MPSA datasets lacked information on specific splice isoforms produced by each variant assayed. For six of the seven MPSA datasets, the splicing assay scores were reported as the difference in the level of splicing (e.g. delta PSI) between the reference and variant constructs [17–19] or as an allelic ratio [21–23], and assay-specific thresholds determined whether or not a variant was considered to alter splicing (Additional file 1: Table S2). It should be noted that, the Vex-Seq and MFASS assays were limited to detecting exon skipping/inclusion, and at least three of the assays would (potentially) misclassify some splicing events as normal (Fig. 1D). Further, although assay scores were intended to provide a measure of splicing impact, outputs from all assays did not differentiate between the types of splicing aberration detected.

The remaining dataset by Patel and colleagues used a Fisher’s exact test of the proportion of reads supporting “no splicing”, “normal splicing” and “aberrant splicing*”* produced from the variant construct compared to the WT construct with “splice-affecting” variants defined by a p < 0.001 [20]. More detailed review of the study of Patel et al. [20] revealed several issues that may compromise the clinical utility of their published assay results. The exon-intron-exon construct design could detect splice site loss or gain induced by splice region variants, but would be less likely to recapitulate the splicing aberrant transcripts in vivo. For example, this design could not detect variant-induced exon skipping (Fig. 1A). The absence of splice donor/acceptor ± 1,2 dinucleotide variants in the assay precluded their use for assay readout calibration, and a previous publication [52] did not show data to support their report of 100% concordance for 10 positive control variants known to alter splicing. To verify concordance of predicted spliceogenic variants with splice assay results, we recalculated the p values using the Fisher’s exact test with normalised counts as described by the authors, but could not replicate the findings using the authors’ selected threshold for splice-affecting variants; 12 variants would have had different classification with our analysis.

**Fig. 2:**
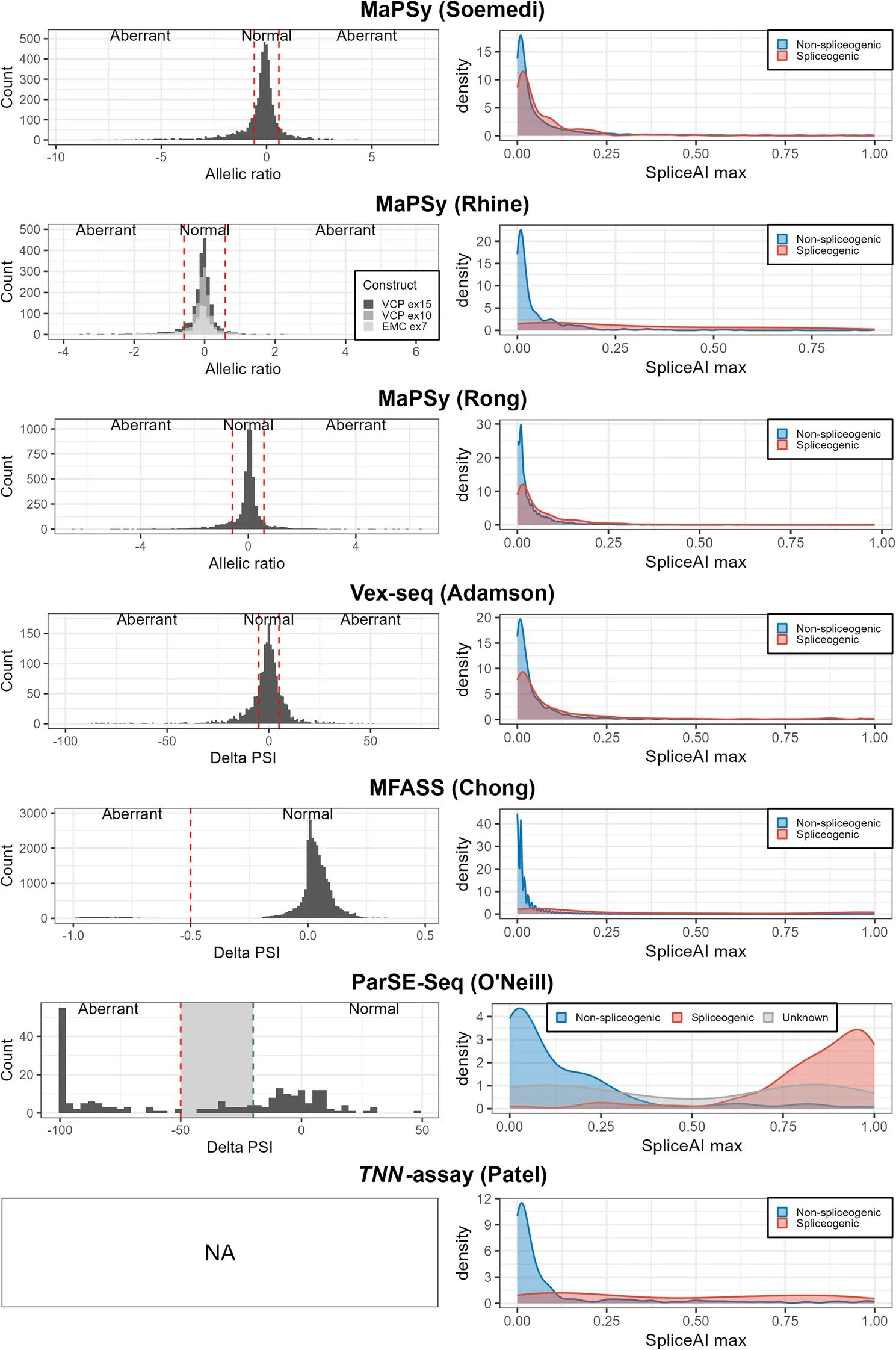
Distribution of SpliceAI scores and MPSA assay data. Left) Histograms of study-reported assay results with the respective thresholds (red dashed lines) used for determining aberrant splicing. The three MaPSy and Vex-seq assays identified variants that impacted splicing that increased or decreased normal exon inclusion. Additionally, Rhine et al., used multiple constructs with different common exons designated by the authors as having different splice site strength (*VCP* exon 15 [Strong], *EMC* exon 7 [Intermediate], or *VCP* exon 10 [Weak]). O’Neill et al., suggested a threshold for no impact of splicing (green dashed line) and a grey zone of ‘intermediate’ impact (grey shaded box). No histogram was possible for the *TTN*-assay as the study as a quantitative assay score was not used. Right) Density plots of SpliceAI max delta scores for assay-defined spliceogenic (red), non-spliceogenic (blue) and unknown impact on splicing (grey) variants

To reliably use splicing data generated by MPSA for variant curation, the assay scores from spliceogenic variants should be distinct from the distributions of non-spliceogenic variants. Only two studies [18, 19] had a multimodal distribution (Hartigan’s *p* <2 × 10^− 16^) of assay scores that were separated by thresholds defined by the study (Fig. 2). Additionally, O’Neill et al. defined three categories to distinguish “abnormal”, “indeterminate” and “normal” impact on splicing for the ParSE-seq dataset (Fig. 2).

As a second approach to assess the assay reliability, correlation of SpliceAI predictions with assay-defined spliceogenic and non-spliceogenic variants was performed (Fig. 2). Only data from ParSE-seq correlated well with SpliceAI predictions: spliceogenic variants reported high SpliceAI scores (mean = 0.79) and non-spliceogenic variants reported low SpliceAI scores (mean = 0.14). “Indeterminate” variants from ParSE-seq had a bimodal SpliceAI score distribution with variants at each end of the spectrum. All other MPSA studies had notable overlap of SpliceAI score distributions between reported spliceogenic and non-spliceogenic variants, suggesting misclassification. ParSE-seq also showed that 99% of the variants with abnormal splicing readout had SpliceAI score ≥ 0.2, the spliceogenicity threshold defined in Walker et al. [8], while the other MPSA studies had lower proportions ranging from 9% to 66% (Additional file 1: Table S6).

**Fig. 3:**
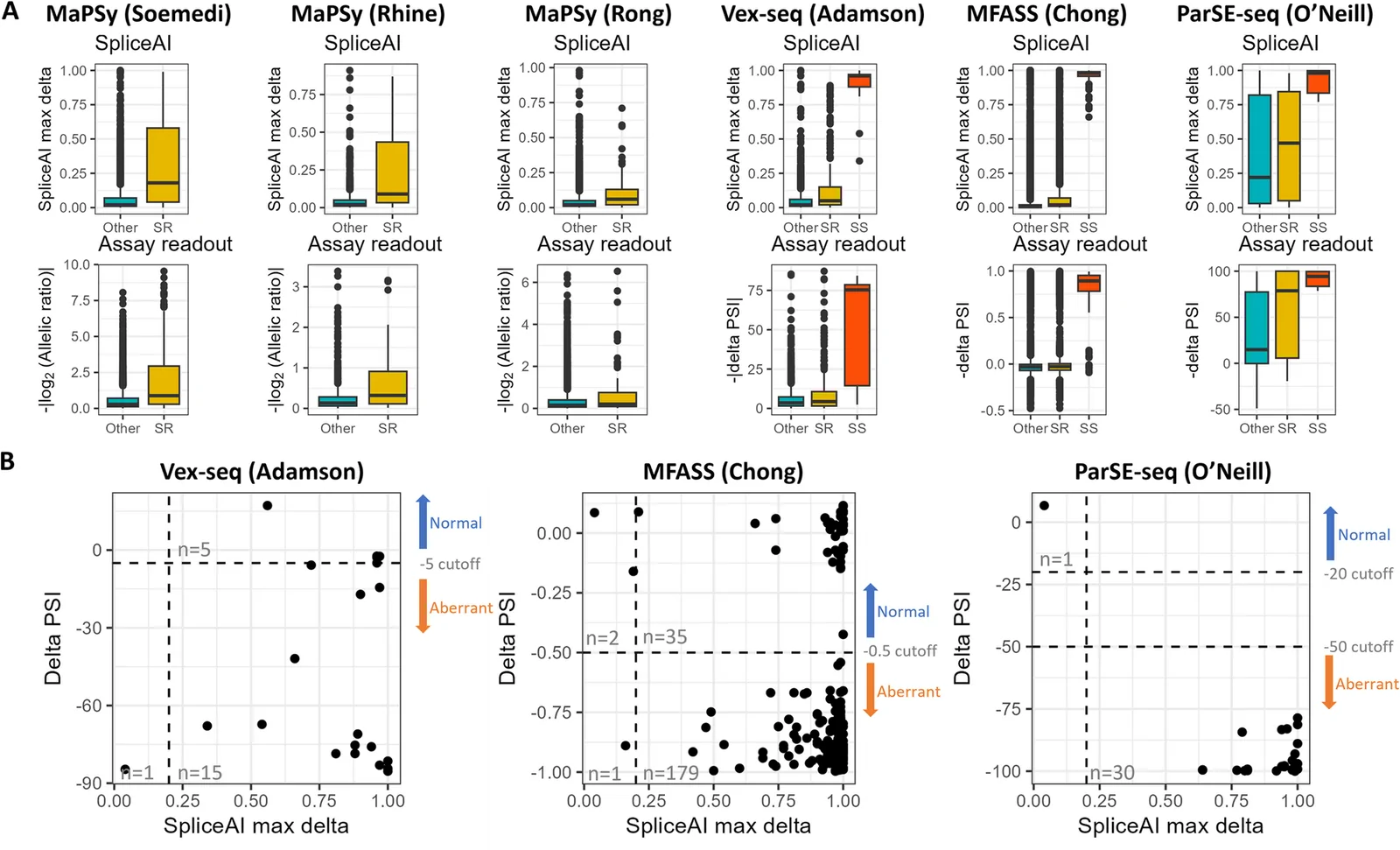
Evaluation of reported splice outcomes for variants at splice motifs. **A** SpliceAI max delta score (top row) and assay readout (bottom row) for variants at the ± 1,2 dinucleotides (red, SS), at splice region (gold, SR), as defined in Walker et al., 2023 [8], and all other sites (teal). **B.** The delta percent spliced in (PSI) and SpliceAI max delta scores for splice donor/acceptor ± 1,2 dinucleotide variants tested by Adamson (left, Vex-Seq) [17], Chong (middle, MFASS) [18] and O’Neill (right, ParSE-seq) [19]. Vertical dashed lines is the SpliceAI max delta score for predicted splicing impact ( > = 0.2). Horizontal dashed lines are the assay-specific thresholds used to define aberrant and normal splicing

To evaluate the suitability of MPSA data for clinical application, we assessed whether each MPSA categorized variants as spliceogenic based on prior expectations, namely variant location in relation to splice donor and acceptor motifs, and SpliceAI score. An overview of findings is summarized in Additional file 2: Fig. S2. Spliceogenic variants with predicted impact were enriched within the splice site motifs as expected, particularly at the splice donor/acceptor ± 1,2 dinucleotide positions (Fig. 3, Additional file 2: Fig. S2). For those studies that assessed variants located within the splice donor/acceptor ± 1,2 dinucleotide positions [17–19], despite enrichment in spliceogenic variants, comparison of reported splice impact against SpliceAI predictions suggested differences in performance across the three methods (Fig. 3). For ParSE-seq, 97% (30/31) of splice donor/acceptor ± 1,2 dinucleotide variants were spliceogenic with only one variant (*SCN5A* c.3957_3963 + 1dup) observed not to impact splicing; the SpliceAI score predicted no splicing impact, highlighting the accuracy of ParSE-seq for the splice donor/acceptor ± 1,2 dinucleotides. For Vex-seq, only 76% (16/21) of splice donor/acceptor ± 1,2 dinucleotide variants impacted splicing, and six variants had splicing assay results discordant with SpliceAI predictions (five predicted spliceogenic but with no observed splicing impact). For MFASS, 83% (180/217) of splice donor/acceptor ± 1,2 dinucleotide variants impacted splicing, with 35 (19%) of these ± 1,2 dinucleotide variants predicted as non-spliceogenic (SpliceAI score < 0.1).

### Traditional minigene assay dataset

**Fig. 4:**
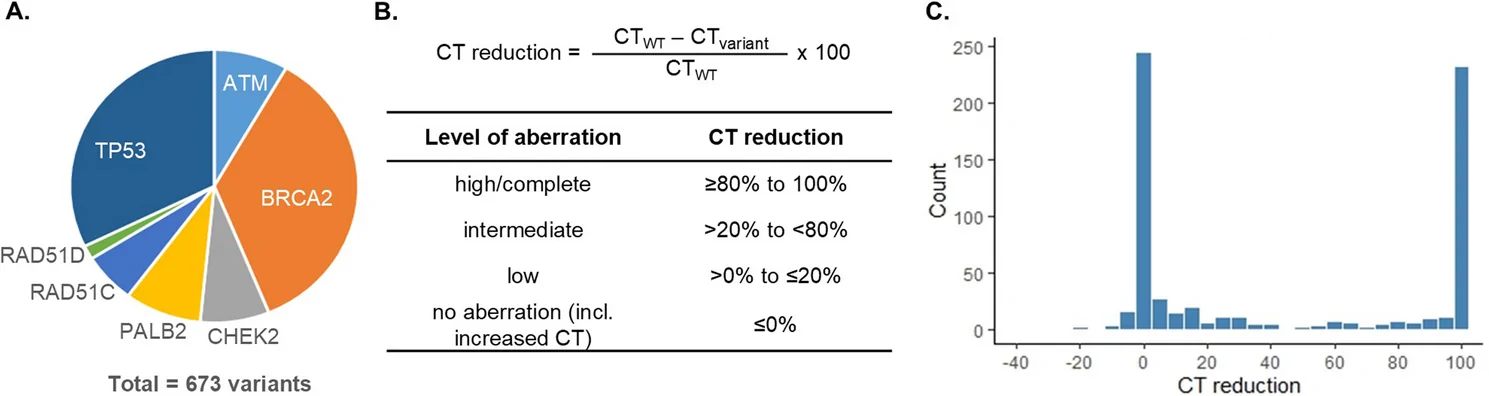
Distribution of variants in the traditional minigene assay dataset and their splicing aberration levels. **A** Proportion of variants across the seven cancer susceptibility genes included in the dataset, *n* = 673. **B** Levels of aberration categorized based on canonical transcript (CT) reduction. **(C)** Distribution of variants based on CT reduction score

The dataset consisted of 600 single nucleotide variants (SNVs) and 73 indels from seven cancer susceptibility genes (Fig. 4A): 359 were exonic, 307 were intronic, and seven were indels spanning the exonic and intronic regions. Of the 673 variants, 23% (135 SNVs and 20 indels) were located at or disrupted the splice donor/acceptor ± 1,2 dinucleotide positions. Seven of the 16 WT minigene constructs produced 100% canonical transcript, and the other nine exhibited background alternative splicing (Additional file 1: Table S7). To account for background splicing, we normalised the level of canonical transcript induced by all variants by calculating the CT reduction (Fig. 4B). CT reduction distribution showed that the vast majority of variants resulted in either no or complete aberration (Fig. 4C). Specifically, 37% (252/673) of the variants resulted in no aberration or increased the canonical transcript level, while 63% (421/673) had splicing impact (82 low, 73 intermediate, and 266 high/complete) (Additional file 1: Table S3). All but one of the splice donor/acceptor ± 1,2 dinucleotide variants showed a high/complete aberration (≥ 80% to 100% CT reduction). The *PALB2* c.108 + 2T > C variant showed low impact on splicing with evidence of alternative splicing in < 15% of transcripts. Notably, this variant resulted from a donor GT > GC substitution and therefore retains the ability to be processed by the U2-type spliceosome [53].

### Evaluation of SpliceAI performance for predicting level of aberration

**Fig. 5:**
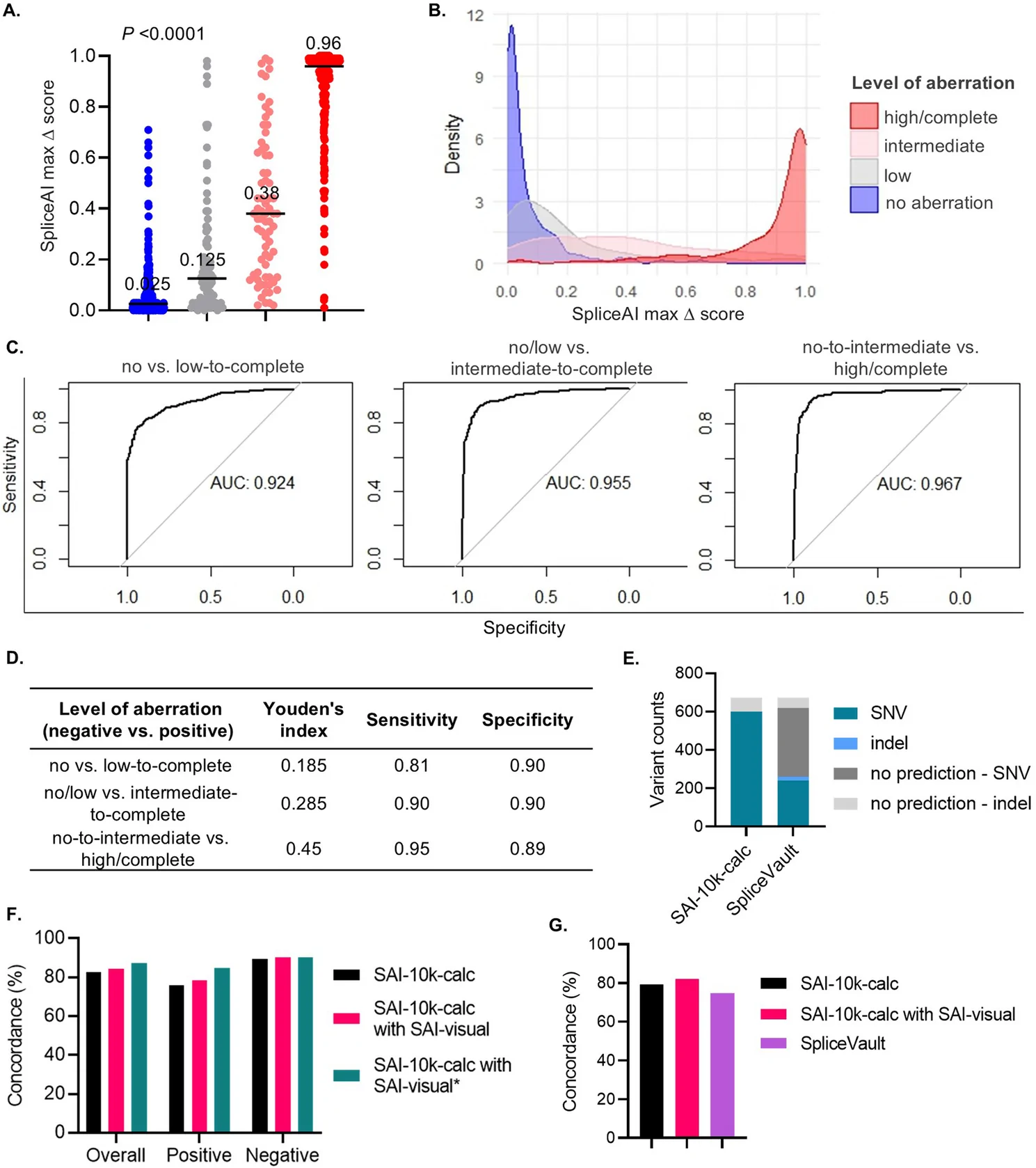
Evaluation of computational tool performance for predicting the level and type of splicing aberration. **(A)** Distribution of SpliceAI max delta score for variants in the different aberration categories: no (blue; Canonical transcript [CT] reduction ≤ 0%), low (gray; CT > 0% to ≤ 20%), intermediate (pink; >20% to < 80%), and high/complete (red; ≥80% to 100%). Median SpliceAI scores are represented by black horizontal lines. **(B)** Density of categorised levels of splicing aberration across the full range of SpliceAI max delta score. **(C)** ROC analysis of SpliceAI max delta score and different levels of aberration. **(D)** Optimal SpliceAI max delta thresholds (Youden index) for separating different levels of aberration. **(E)** Number of variants with transcript prediction (shades of blue) and without prediction (shades of gray) by SAI-10k-calc and SpliceVault, *n* = 673. SAI-10k-calc returned positive and negative predictions for SNVs only. SpliceVault returned positive predictions only for SNVs and indels, nearly all located in the splice region. **(F)** Concordance of SAI-10k-calc transcript prediction with minigene assay results for all SNVs, *n* = 600. The positive control set included SNVs inducing intermediate to complete aberration, while the negative control set included SNVs with no/low aberration. SAI-10k-calc with SpliceAI-visual inspection of 50 flagged variants slightly increased the concordance. *Inclusion of additional SNVs with concordant functional consequence (PTC or in-frame transcript) further increased the concordance (teal). **(G)** Concordance of positive transcript prediction by SAI-10k-calc and SpliceVault with minigene assay results for SNVs located in the splice donor and acceptor motifs, *n* = 257

Our analysis of the traditional minigene dataset showed that SpliceAI score for a variant generally increases with the categorized levels of variant-induced splicing aberration (Fig. 5A and B). There was a significant difference in SpliceAI scores (Kruskal-Wallis *p* < 0.0001) across the no (median = 0.025), low (median = 0.125), intermediate (median = 0.38), and high/complete (median = 0.96) aberration categories. Moreover, ROC analysis showed that SpliceAI can distinguish between different aberration level categories with good accuracy (Fig. 5C and D). The optimal thresholds to predict different ranges of aberration levels were: 0.185 for “no versus low-to-complete” (AUC = 0.924), 0.285 for “no/low versus intermediate-to-complete” (AUC = 0.955), and 0.45 for “no-to-intermediate versus high/complete” (AUC = 0.967). Importantly, the 0.285 threshold showed 90% sensitivity and 90% specificity for separating no/low versus intermediate-to-complete aberration, while the 0.45 threshold achieved 95% sensitivity and 89% specificity for separating high/complete from no-to-intermediate aberration (Fig. 5D).

We then tabulated results to assess the proportion of variants falling into the different aberration level categories for different SpliceAI score bins (Additional file 1: Tables S8 and S9), some selected to capture thresholds recommended previously e.g. <0.05, 0.1, 0.2, 0.5 and 0.8 [8, 40, 54]. For the 518 variants located outside the splice donor/acceptor ± 1,2 dinucleotide positions, there was convincing evidence for a correlation between SpliceAI score and level of aberration (τ = 0.66, *p* = 3.2 e-72) (Table 1, Additional file 1: Table S8). Of the spliceogenic variants with SpliceAI score ≤ 0.05, 78% had low level impact and only 8% (3/26) had high/complete impact. For the latter, the results for two of three variants are indicative of impact via splicing regulatory elements which remain poorly predicted by SpliceAI and other tools [43, 54]. In contrast, for spliceogenic variants with SpliceAI score between ≥ 0.2 and < 0.5, 24% had low, 50% had intermediate, and 26% had high/complete aberration. At SpliceAI score ≥ 0.5, 72% of spliceogenic variants had high/complete aberration: 56% for those from ≥ 0.5 and < 0.8, and 83% for those with score ≥ 0.8. Notably, no variants with SpliceAI score ≥ 0.8 were shown to be non-spliceogenic in this dataset. Unsurprisingly, the correlation was even more striking when analyses included variants located at the splice donor/acceptor ± 1,2 dinucleotide positions (Additional file 1: Table S9), the vast majority of which had SpliceAI score ≥ 0.8 (τ = 0.76, *p* = 1.6e-121).

**Table 1:** Distribution of variants outside the splice donor/acceptor ± 1,2 dinucleotide positions across different ranges of SpliceAI max delta scores

SpliceAI max delta score	No. of variants					Proportion in the spliceogenic set		
	Non-spliceogenic set	Spliceogenic set^a^	Low	Intermediate	High/complete	Low	Intermediate	High/complete
≤ 0.05	167	36	28	5	3^*b*^	0.78	0.14	0.08
> 0.05 & ≤0.1	31	14	7	7	0	0.50	0.50	0.00
> 0.1 & <0.2	30	32	22	9	1	0.69	0.28	0.03
≥ 0.2 & <0.5	17	54	13	27	14	0.24	0.50	0.26
≥ 0.5 & <0.8	7	52	7	16	29	0.13	0.31	0.56
≥ 0.8	0	78	4	9	65	0.05	0.12	0.83
TOTAL =	252	266	81	73	112			

^a^The spliceogenic set is subdivided into three levels of splicing impact based on CT reduction - low (> 0% to ≤ 20%), intermediate (> 20% to < 80%), and high/complete (≥ 80% to 100%). ^b^Two of the three false-negatives (*BRCA2* c.441 A > G and c.451G > A) were located outside the donor/acceptor motifs and led to (multi-)exon skipping, indicating effect on splicing regulatory elements; the remaining false-negative (*PALB2* c.3113 + 3 A > G) resulted in donor loss leading to cryptic donor activation and exon skipping. Correlation between SpliceAI score and level of aberration: τ = 0.66, *p* = 3.2 e-72

For variants outside the splice donor/acceptor ± 1,2 dinucleotide positions, further stratification of the bins for scores ≥ 0.2 indicated that, based on similarity of proportions in the spliceogenic set for different bin strata, there was slightly improved correlation between SpliceAI score and level of aberration when scores were binned as: ≥0.2 & <0.3; ≥0.3 & <0.4; ≥0.4 & <0.75; ≥0.75 (τ = 0.66, *p* = 1.4e-73; Additional file 1: Table S9).

### Calibration of SpliceAI for refined ACMG/AMP computational code application using quantitative traditional minigene assay data

The ClinGen Splicing Subgroup [8] previously performed a calibration to demonstrate the utility of SpliceAI to provide evidence towards or against spliceogenicity for variants located outside of the splice donor/acceptor ± 1,2 dinucleotide positions, providing the basis for selecting score thresholds for conservatively assigning computational codes BS4 and PP3. However, this previous analysis only considered whether a variant may result in aberrant splicing since information about the level of the variant-induced event/s was either lacking or measured heterogeneously across the different studies forming the reference datasets.

Building on the findings above (Table 1; Additional file 1: Tables S8 and S9), we performed LR analysis to estimate the evidence strength applicable for SpliceAI score thresholds to distinguish different ranges of splicing aberration levels. Results are summarized in Table 2. Using “no aberration” as the negative truthset, and thresholds previously shown to yield moderate evidence towards or against spliceogenicity [8], the LR for a SpliceAI score threshold of ≥ 0.2 for predicted aberrant splicing equated to a moderate evidence for spliceogenicity (low to complete aberration), score > 0.1 and < 0.2 equated to indeterminate evidence, but score ≤ 0.1 equated to supporting evidence for no aberration. Given that this semi-quantitative data would be expected to place previously undetected “low” level events in the positive reference set, we estimated LRs using alternative score thresholds and impact-level groups. Altering the lower threshold to ≤ 0.05 gave an LR equating to moderate evidence for no versus low-to-complete aberration, with > 0.05 and < 0.02 providing no evidence. Then, since there is data to indicate that low level of aberrant splicing (≤ 20% expression) is very unlikely to confer pathogenicity in the context of hereditary cancer genes at least [55, 56], while intermediate level aberrant splicing is adequate to confer pathogenicity for at least some genes [26, 57], we compared no/low as the negative reference set versus intermediate-to-complete aberration as the positive control set. Our analysis showed that SpliceAI score threshold of ≥ 0.3 provides moderate evidence for intermediate to complete aberration, and score ≤ 0.2 provides moderate evidence for no/low aberration. These thresholds showed 82% sensitivity and 86% specificity for predicting intermediate to complete aberration due to variants located outside the splice donor/acceptor ± 1,2 dinucleotide positions.

**Table 2:** Likelihood ratio analysis of the SpliceAI max delta score for variants outside the splice donor/acceptor ± 1,2 dinucleotide positions

SpliceAI max delta score	Negative set^a^		Positive set^a^		LR	Low CI	High CI	Evidence strength^b^
	*n*	Proportion	*n*	Proportion				
no aberration vs. low-to-complete aberration using score thresholds from previous calibration								
≤ 0.1	198	0.79	50	0.19	0.24	0.18	0.31	Supporting (no aberration)
> 0.1 & <0.2	30	0.12	32	0.12	1.01	0.63	1.61	Indeterminate
≥ 0.2	24	0.10	184	0.69	7.26	4.92	10.72	Moderate (low-to-complete aberration)
TOTAL =	252		266					
no aberration vs. low-to-complete aberration, using score thresholds set for this semiquantitative dataset								
≤ 0.05	167	0.66	36	0.14	0.20	0.15	0.28	Moderate (no aberration)
> 0.05 & <0.2	61	0.24	46	0.17	0.71	0.51	1.01	Indeterminate
≥ 0.2	24	0.10	184	0.69	7.26	4.92	10.72	Moderate (low-to-complete aberration)
TOTAL	252		266					
no/low aberration vs. intermediate-to-complete aberration, using score thresholds set for this semiquantitative dataset								
≤ 0.2	287	0.86	26	0.14	0.16	0.11	0.23	Moderate (no/low aberration)
> 0.2 & <0.3	13	0.04	8	0.04	1.11	0.47	2.62	Indeterminate
≥ 0.3	33	0.10	151	0.82	8.24	5.92	11.47	Moderate (intermediate-to-complete aberration)
TOTAL	333		185					
no-to-intermediate aberration vs. high/complete aberration, using score thresholds set for this semiquantitative dataset								
≤ 0.4	353	0.87	12	0.11	0.12	0.07	0.21	Moderate (no-to-intermediate aberration)
> 0.4 & <0.5	10	0.02	6	0.05	2.18	0.81	5.86	Supporting (high/complete aberration)
≥ 0.5	43	0.11	94	0.84	7.92	5.91	10.63	Moderate (high/complete aberration)
TOTAL	406		112					

Abbreviation: *LR* likelihood ratio, *CI* confidence interval, *n* number

^a^The negative and positive sets were adjusted according to the level of aberration being tested. Data drawn from traditional minigene assay dataset only

^b^Criteria thresholds as defined in Tavtigian et al., [49]

### Evaluation of SAI-10k-calc and SpliceVault performance for predicting aberration type

Of the 673 variants, 600 were SNVs enabling computational splicing assessment with SAI-10k-calc (Fig. 5E), which automatically predicts the transcript type based on the combination of SpliceAI scores. For this assessment, we designated the variants with intermediate-to-complete aberration as the positive control set (*n* = 299), and no/low aberration as the negative control set (*n* = 301). This grouping was selected based on results shown in Table 2, where the proportion of true positives (82%) and true negatives (86%) using SpliceAI score thresholds ≤ 0.2 and ≥ 0.3 were markedly higher than true positives (69%) and true negatives (79%) observed using previously recommended thresholds (≤ 0.1 and ≥ 0.2) from calibration using non-quantitative RNA findings. Detailed descriptions of concordance and discordance between predicted and observed transcripts are provided in Additional file 1: Tables S3 and S10. SAI-10k-calc aberrant transcript prediction for SNVs was 83% (496/600) concordant with minigene assay results (Fig. 5F). Another 8% (50/600) of the SNVs were flagged by SAI-10k-calc and underwent manual inspection using SpliceAI-visual [44] to obtain the final transcript prediction (see Methods). SAI-10k-calc transcript prediction with additional SpliceAI-visual manual inspection achieved a slightly greater overall concordance of 84% (506/600) (Fig. 5F); concordance was 79% (235/299) for positive prediction of aberration (as defined above, at least intermediate (> 20%) expression level), and 90% (271/301) for negative prediction of aberration (no/low aberration). For 18/27 variants assigned as discordant or partial concordance, where the predicted aberrant transcript was absent or expressed at low level and other dominant transcript/s were detected in the assay (coded as 3 + and 4 + in Additional file 1: Table S10), the predicted and observed transcripts had the same functional consequence (PTC or in-frame); considering these additional 18 variants with concordance in functional consequence, SAI-10k-calc overall concordance increased to 87% (524/600) (Fig. 5F).

We also assessed the concordance of SpliceVault [45] Top-4 alternative splicing events with minigene assay results (Additional file 1: Table S3). The SpliceVault VEP plugin returned Top-4 events for 39% (261/673) variants, including 242 SNVs and 19 indels (Fig. 5E); of these, 254 variants were located in the splice donor and acceptor motifs. Since SpliceVault predicts the alternative splicing events including effect on reading frame chiefly when the native splice site motif is abrogated, we calculated the concordance of SpliceVault Top-4 using 291 positive controls (257 SNVs, 34 indels) located in the splice donor and acceptor motifs only. SpliceVault Top-4 events matched with at least one transcript with > 20% expression for 75% (192/257) of the positive control SNVs (Fig. 5G) and 50% (17/34) of the indels. All variants returning SpliceVault predictions had SpliceAI score ≥ 0.2. For the same set of 257 positive control SNVs, we observed greater concordance for SAI-10k-calc alone (79%) and SAI-10k-calc with SpliceAI-visual (82%) (Fig. 5G).

### Comparison of traditional minigene and patient RNA results

**Fig. 6:**
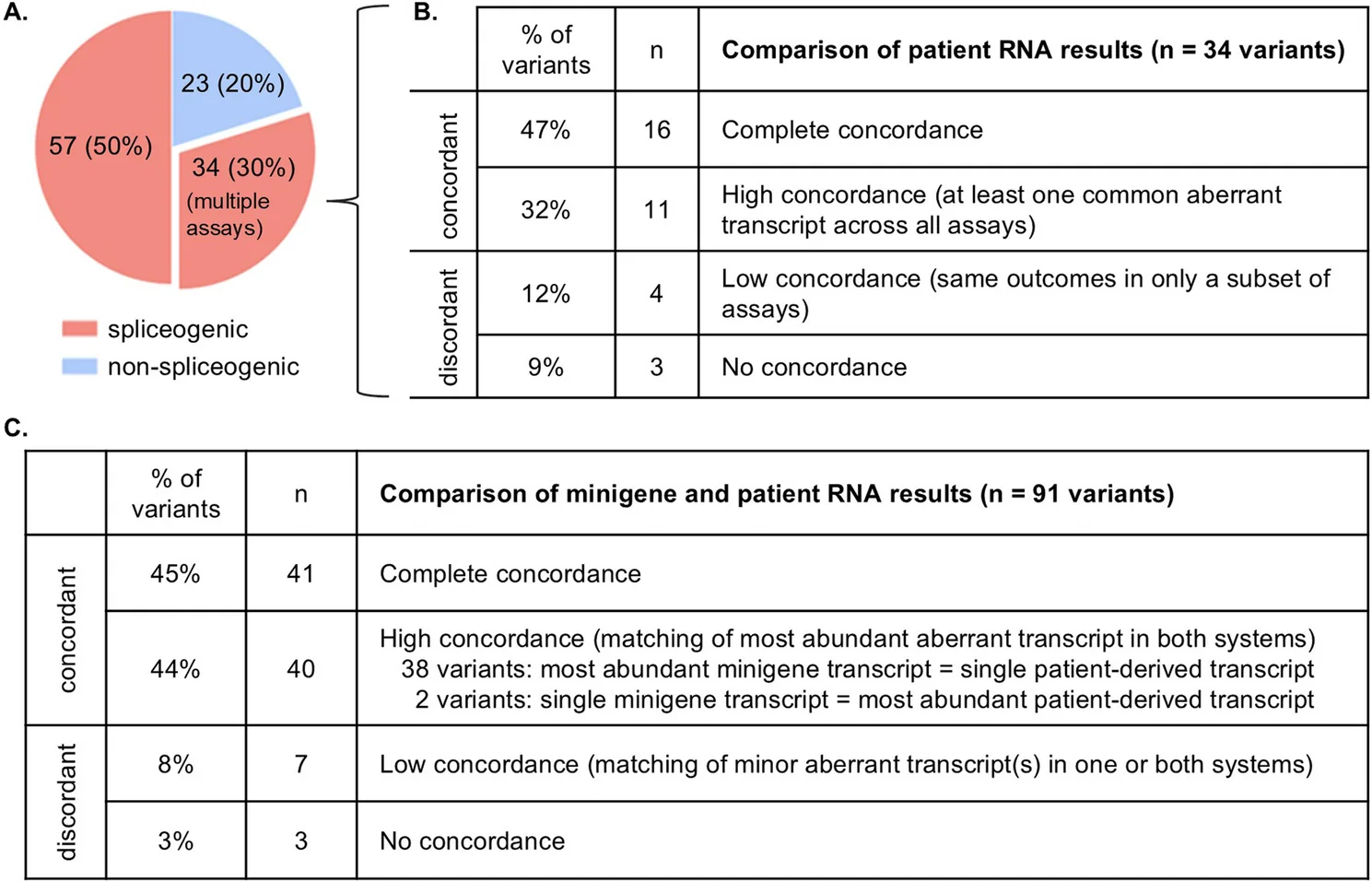
Comparison of traditional minigene and patient RNA results. **A** Number of variants with published patient RNA splicing assay results (*n* = 114), drawn from 52 publications (Additional file 1: Tables S11 and S12). **B** Comparison of patient RNA results for 34 variants assayed in multiple studies and shown to alter splicing in at least one study. **(C)** Comparison of minigene and patient RNA results for 91 variants that demonstrated altered splicing in at least one patient RNA assay

Our literature search identified 114 variants in the traditional minigene assay dataset with published patient RNA splicing assay results (Fig. 6A), of which 91 variants demonstrated impact on splicing in patient assays (Additional file 1: Table S11) and 23 variants did not (Additional file 1: Table S12). Patient assays were conducted without NMD inhibition for 55%, only with NMD inhibition for 28%, and with or without NMD inhibition across different studies for 17%. For the 34 variants assayed in multiple patient RNA studies, 47% showed complete concordance, 32% high concordance, 12% low concordance (consistent outcomes in only a subset of assays), and 9% no concordance across all assays (Fig. 6B). Variability across patient RNA results for the same variant could be ascribed to methodological issues, in particular failure to detect transcripts due to lack of NMD inhibition or detection limits (Additional file 1: Table S11). Patient genomic context such as common polymorphisms may also cause upregulation of naturally occurring alternative transcripts, which could contribute to patient RNA results variability.

For variants with impact on splicing from the patient RNA assays, comparison of minigene and patient-derived RNA results revealed a high level of agreement across all seven genes (Fig. 6C, Additional file 1: Table S11). Overall, 89% of variants had concordant splicing outcomes (45% complete concordance, and 44% high concordance where the predominant transcripts agreed across the two systems). The remaining 11% were discordant, comprising 8% with low concordance (where agreement was limited to minor transcripts only) and 3% with no concordance.

Among the 40 highly concordant cases, two involved a single aberrant minigene transcript matching with the most abundant patient-derived transcript, and 38 had a single patient-derived transcript matching the most abundant transcript detected in the minigene assay. Of the latter, the minigene assay revealed additional transcripts in comparison to patient RNA assays, either lowly expressed (≤ 20% of the total RNA pool, *n* = 30) or moderately expressed transcripts (23–48%, *n* = 8) that were likewise absent in patient RNA assays.

Possible explanations for the observed discrepancies between the minigene and patient findings (no/low concordance) are detailed in Additional file 1: Table S11. In general, the splicing outcome pattern likely reflects the greater sensitivity of the minigene semi-quantification method in detecting low-abundance splice products as these are probably missed in patient RNA assays using agarose gel electrophoresis and Sanger sequencing. In addition to the detection limit, agarose gel lacks the resolution to distinguish transcripts that differ by only a few nucleotides from the full-length transcript and therefore may not be flagged for further characterisation. Moreover, most of the patient RNA assays were conducted without NMD inhibition, which likely contributed to the absence of certain PTC-containing transcripts that were detected in the minigene assay under NMD-inhibited conditions. Other possible explanations include differences in the location of primers, alternative splicing specific to leukocytes or lymphoblastoid cell lines from patients, minigene construct design limitation (e.g. removal of gene context important to splicing), and use of different techniques and reagents.

For *BRCA2*, where comprehensive splicing assay data were available through our in-house database, assay outcomes indicating no aberration also demonstrated high agreement with patient RNA data. Of the 23 *BRCA2* variants with no aberration in patient RNA assays (Additional file 1: Table S12), 22 (96%) showed no/low aberration in the minigene assay. One variant (*BRCA2* c.441 A > G), showed complete splicing impact (100% expression of PTC-containing transcripts) in the minigene assay but no detectable impact in the assay of patient RNA from blood without NMD inhibition [10]. This variant has a low SpliceAI score of 0.04; however, it was previously identified to lie within an exonic splicing enhancer motif based on microdeletion analysis [34]. In addition to possible degradation of PTC-containing transcripts in the patient sample, differences in splicing factor activity between the minigene system and blood may explain the discordance.

These findings indicate that the minigene assay reliably recapitulates patient-derived splicing patterns in most cases, with discrepancies occurring primarily in low-abundance splicing events.

### Clinical calibration to derive evidence strength applicable for construct-based RNA results

Clinical calibration of the collated minigene dataset showed that variants leading to high (≥ 80%) or low (≤ 20%) expression of transcripts with predicted LOF consequence provide strong evidence towards or against pathogenicity, respectively (Table 3). Evidence strength was unchanged after exclusion of higher-scoring variants at splice donor/acceptor ± 1,2 dinucleotide positions (Table 3). Given the far lower confidence in the evidence strength estimate for the remaining two categories (> 20 and < 50; ≥50 and < 80), it is suggested to apply no evidence for these categories based on the current findings. Sensitivity analysis limiting the B/LB reference set to variants with at least one B classification, and the P/LP reference set to variants with at least one P classification, did not alter findings (data not shown).

**Table 3:** Clinical calibration of traditional minigene assay results considering levels of predicted LOF transcripts

Percentage of predicted LOF transcripts	B/LB reference set		*P*/LP reference set		LR	Low CI	High CI	Evidence strength based on LR	Suggested evidence strength considering CI	Points
	*n*	Proportion	*n*	Proportion						
Calibration including variants at splice donor/acceptor ± 1,2 dinucleotide positions										
≤ 20	76	0.92	1	0.01	0.01	0.00	0.05	Benign_strong	Benign_strong	-4
> 20 & <50	5	0.06	2	0.01	0.20	0.04	1.01	Benign_moderate	No evidence	0
≥ 50 & <80	1	0.01	7	0.04	3.50	0.44	27.98	Pathogenic_supporting	No evidence	0
≥ 80	1	0.01	156	0.94	78.00	11.11	547.44	Pathogenic_strong	Pathogenic_strong	+ 4
TOTAL =	83		166							
All reference set variants, *n* = 249										
Calibration excluding variants at splice donor/acceptor ± 1,2 dinucleotide positions										
≤ 20	76	0.92	0	0.00	0.02	0.00	0.30	Benign_strong	Benign_strong	-4
> 20 & <50	5	0.06	0	0.00	0.26	0.02	4.62	Benign_supporting	No evidence	0
≥ 50 & <80	1	0.01	1	0.04	2.96	0.19	45.84	Pathogenic_supporting	No evidence	0
≥ 80	1	0.01	27	0.96	80.04	11.39	562.24	Pathogenic_strong	Pathogenic_strong	+ 4
TOTAL	83		28							
All reference set variants, *n* = 111										

Abbreviation: *B* Benign, *LB* Likely Benign, *P* Pathogenic, *LP* Likely Pathogenic, *LR* likelihood ratio, *CI* confidence interval; n, number

## Discussion

We conducted a comprehensive evaluation of splicing assay datasets from MPSAs (7 datasets, 41,128 variants in total) and traditional minigene assays (14 selected datasets, 673 variants in total), and summarise below how the findings may inform recommendations for the application of construct-based splicing assay and bioinformatic prediction data for clinical variant classification.

### Important considerations related to use of MPSA data for clinical variant classification

Our analysis of MPSA datasets ranging from gene-specific assays (ParSE-seq and *TTN* assay) to broad MPSA screens (MaPSy, Vex-seq, and MFASS) has highlighted the need for rigorous evaluation of design and performance for their use in clinical variant classification. While specificity measures (Additional file 1: Table S6) for all MPSA studies does suggest these were accurate at identifying variants that did not impact splicing, this may reflect the fact that most variants assayed (87%) were located outside the splice region where variation has low prior probability to alter splicing.

The MPSAs all lacked information on specific alternatively spliced transcripts produced by each variant assayed; this absence of detailed characterisation of aberrant transcripts in MPSAs prevents the determination of effect on the amino acid sequence, a crucial factor for predicting the functional consequence of a spliceogenic variant, and thus its likelihood to cause disease. All MPSA datasets were also limited by assay design, restricting detection to specific splicing events and thereby capturing only a subset of RNA alterations (Fig. 1D; Additional file 1: Table S3). Even considering the assay design limitations, the assay scores generated by five of seven MPSA datasets (MaPSy (3 datasets), Vex-seq, and *TTN* assay) were not able to clearly differentiate aberrant splicing events from normal splicing events, based on both overall distribution and correlation of reported splicing impact with SpliceAI score (Fig. 2). Notably, our scrutiny of assay data for splice donor/acceptor ± 1,2 dinucleotide variants with high probability to impact splicing revealed that a considerable number had no observed splicing impact for the Vexseq (24%) and MFASS (17%) datasets. In summary, all these assays exhibited significant experimental noise that contributed to the uncertainty of assay results. For Vex-seq and MaPSy, this was previously highlighted by The Critical Assessment of Genome Interpretation Consortium [58]. For MaPSy, this noise may be in part due to the assay normalisation. MaPSy used the variant sequence to de-multiplex and could only measure reads that mapped to normal exon inclusion. These counts were normalised to the DNA input (the amount of minigene added into the experiment) and makes the large assumption that the transfection efficiency and transcription rate of each minigene for each cell will be equal.

In contrast, the ParSE-seq dataset showed all the characteristics of a well-performing assay, with a bimodal distribution, expected correlation with SpliceAI score, and complete concordance between observed aberration and prediction of splicing impact for variants at the splice donor/acceptor ± 1,2 dinucleotide positions. This likely reflects that this *SCN5A* gene-specific assay offered a key experimental advantage that was absent in broad MPSA screens. The ParSE-seq minigene constructs included longer flanking intronic sequences and thus are expected to have captured more intronic splicing signals than the minigene constructs used in broad MPSA screens. In addition, the study design itself allowed for assessment of assay performance: the ParSE-seq approach utilized two cell lines, one of which was physiologically relevant; validation was carried out for selected variants using orthogonal methods; the clinical validity of the assay was evaluated by including ClinVar P/LP and B/LB variants as validation controls. The ClinVar controls formed the basis for clinical calibration of the assay according to the framework developed by Brnich et al. (2019) [59], reaching a strong level of evidence towards or against pathogenicity. However, we note that the subsequent application of RNA-based evidence for clinical variant classification as part of the study by O’Neill et al. [19] did not openly consider aberration type and effect on reading frame, and assumed that all variants that induced abnormal splicing had deleterious biological consequences. Characterisation of the aberrant transcript(s) produced by each variant assayed was not included in the published dataset to confirm this assumption.

We highlight that any inferences (including benchmarking) from the six MPSA datasets we demonstrated to have poor performance should be viewed with caution. For example, AlphaGenome used the MFASS dataset to benchmark its algorithm against SpliceAI and other splicing predictors [60], and demonstrated poor performance for all predictors (AUC < 0.54), in line with our findings. However, the excellent performance of the remaining MPSA dataset [19] does demonstrate that large-scale MPSA datasets can have value for clinical application.

### Value of selected traditional minigene data for informing clinical variant classification

We illustrate that strategically selected traditional minigene assay studies generating semi-quantitative RT-PCR data provide information that can benefit clinical variant classification - either directly as RNA evidence of variant impact or by informing the application of splicing aberration prediction data in classification algorithms. The selection of datasets generated from a single laboratory was intentional, as it allowed us to minimise technical variability and assess traditional minigene assay performance under controlled conditions. All the component studies used multi-exon constructs, sensitive capillary electrophoresis and automated DNA sequencing, performed assays in triplicate, and included WT and known spliceogenic experimental controls. We anticipate that our findings could be generalisable to other well-designed traditional minigene assays. Our previous analysis [8] demonstrating utility of SpliceAI score to predict variant impact on splicing used results generated using a variety of assay types, including traditional minigene assays of variable design from multiple laboratories.

First, we demonstrate that the selected traditional minigene assays perform very well in measuring variant impact on splicing, from comparison of the experimentally observed variant-induced splicing aberration versus those expected based on SpliceAI prediction - using a variety of score thresholds previously recommended to separate variants according to likelihood to impact splicing. This is unsurprising, since the studies were specifically selected because they used multi-exon construct design (ranging from three to nine exons) that delivered good experimental reproducibility due to highly standardised protocols using the same reagents; all assays were performed in a minimum of three replicates, producing results with low standard deviation. We note that, for genes where lower levels of aberrant splicing (e.g. 10–20% expression) are proven to be associated with disease predisposition, other thresholds may be considered relevant for LR estimation and application of bioinformatic evidence of disease-associated splicing aberrations.

Second, constructs composed of three or more exons generally show similar or identical results to those derived from patient RNA (where these are available) when the baseline splicing pattern of the minigene construct has been confirmed to match that observed in patient-derived RNA. As a specific example, variant *BRCA2* c.8488-1G > A showed different splicing outcomes in 2-exon [61] and 9-exon [38] minigenes, with the latter emulating results from patient material. Here, we demonstrate that results from the selected traditional minigene assays were highly concordant with those generated from patient RNA; the single outlier variant (complete impact for construct, no impact for patient RNA) was located within an exonic splicing enhancer motif, with splice impact potentially arising due to the truncated genomic context of the construct and absence of sequence essential for regulating splicing activity in vivo. Moreover, there was considerable variation between different patient RNA results for a given variant, likely due to differences in experimental methods. Assuming that patient RNA results remain the gold-standard as the source of information for variant impact on splicing, these findings indicate that minigene assay results can provide a reliable source of information where “validation” of such assay findings is achieved through faithful replication of results from *well-designed* patient RNA assays for a subset of minigene results. Overall, these results also justify the use of construct-based data as a means to quantify allele-specific expression of aberrant transcripts to supplement patient-derived RNA results.

Third, we have demonstrated through clinical calibration that multi-exon construct-based RNA results, after considering the aberration type and level, can provide strong evidence towards or against pathogenicity. Our calibration findings provide guidance, for hereditary cancer genes at least, regarding the association between level of predicted LOF transcripts and pathogenicity; the LR estimates indicate that more extreme expression level categories (≤ 20% and ≥ 80%) are required to confidently assign evidence against or towards pathogenicity. Although there appeared to be an ordered trend in evidence strength for bins representing increasing levels of predicted LOF transcript/s induced by a variant, statistical power was constrained by the limited number of variants for the inner bins in particular. We believe that larger studies are needed to provide more clarity about the strength of evidence associated with “intermediate” levels of aberrant splicing for hereditary cancer genes. Our findings demonstrate how construct-based studies can inform the level of aberrant splicing associated with disease causality, for application to other gene-disease relationships.

Importantly, we have also shown the extensive value of minigene assay results for informing bioinformatic prediction of both level and type of aberrant splicing induced by a variant, including those located outside of the splice donor/acceptor ± 1,2 dinucleotide positions. Our results provide a baseline for selecting SpliceAI score bin thresholds that provide not only a probability that a variant will be spliceogenic, but how likely the level of splicing will be high-to-complete. This allows more nuanced estimation of the LRs for bioinformatic bins, with potential to inform gene-specific score thresholds selected based on gene-disease knowledge, to separate disease-associated levels of aberrant splicing from those that are tolerated. It is also possible to better utilise SpliceAI predictions by using SAI-10k-calc to predict the variant-induced aberration(s), which in our analysis demonstrated 79% concordance for positive prediction of aberration type. This approach may advance the use of such predictions beyond spliceogenicity to determine pathogenicity (or benignity) through the application of gene-specific knowledge about transcript structure and clinically important protein domains. We note that for SNVs, SAI-10k-calc provided a modestly better prediction of no/low aberration than SpliceAI score threshold ≤ 0.2 (89% versus 87%), likely reflecting that SAI-10k-calc utilises the combination of all four output scores from SpliceAI. Moreover, our results suggest that concordance in functional consequence assigned to predicted transcripts versus observed transcripts may reach as high as 87%, with no material difference in the expected clinical relevance for ~⅔ of variants assigned as partial or complete discordance from our analysis.

Another important outcome of the results from our minigene analysis is their implications for practical application of the PS1 code in the context of predicted splicing impact. Namely, it is possible to infer pathogenicity for a predicted spliceogenic variant based on pathogenic classification for another variant with the same predicted impact on splicing [8]. As currently stated, the prerequisite for applying the PS1 codes is as follows: the predicted event of the variant under assessment must “precisely match the predicted event of the comparison (likely) pathogenic variant (e.g., both predicted to lead to exon skipping, or both to lead to enhanced use of a cryptic splice motif), AND the “strength of the prediction for the variant under assessment must be of similar or higher strength than the strength of the prediction for the comparison (likely) pathogenic variant” [8]. The demonstrated good performance of SAI-10k-calc to predict aberration events provides confidence that it is possible to assess if two different variants have matching predicted events. Further, the observation that SpliceAI score range categories can be used to infer level of variant-induced aberration provides justification that variants falling into the same SpliceAI score bin might be considered to have similar strength of prediction for both variant type and level of aberration. We acknowledge that the actual measurement of aberration level might differ between different assay methods, but this is irrelevant for application of PS1 which is based on comparing predicted splicing impact, and relies on the fact that the comparison pathogenic variant reaches this classification due to consideration of clinical data (as is the situation for applying PS1 in the context of missense variants). Likewise, between-gene differences in the level of aberrant splicing required to confer pathogenicity are accommodated by the fact that PS1 application is restricted to variants in the same splice region of the same gene.

### Using construct-based splicing assay results in variant curation

The use of RNA splicing data from traditional minigene assays as evidence for classification is already being implemented by some ClinGen VCEPs e.g. Criteria Specifications for *BRCA1/2* v1.2, *CTLA4* v1.0, *LDLR* v1.2, *PTEN* v3.1, *RS1* v1.0. Moreover, the InSiGHT Hereditary Colorectal Cancer/Polyposis and ENIGMA *BRCA1* and *BRCA2* VCEPs specifically recognise the value of minigene assays to provide quantitative information for variants located outside of the splice donor/acceptor ± 1,2 dinucleotide positions. RT-PCR assays using multi-exon minigene constructs can measure aberrant and naturally occurring alternative splicing spanning multiple exons, and can also characterise complex splicing aberrations and multiple transcripts arising from one variant allele. Inferring functional consequences, which include potential gain-of-function, from splicing outcomes remain challenging. This limitation is especially important for variants in genes or regions where activating or non-LOF mechanisms are plausible. Minigene assay designs and calibration analyses may not be designed appropriately to distinguish these effects. When well-designed, minigene assays can precisely define the type and level of splicing aberration, including whether the effect is complete or partial, its impact on the reading frame, the affected functional domains, and the presence of rescue transcripts from alternative splicing. That is, traditional minigene splicing assay studies offer a considerable level of detail in the characterisation of splicing products. They also provide an avenue to provide gene-specific recommendations on the minimum level of aberrant splicing that is associated with disease presentation. Altogether, a well-validated assay can generate all information necessary to assign an ACMG/AMP evidence code according to a decision flowchart proposed by the ClinGen Splicing Subgroup, which allows weights up to PVS1 to be assigned based on RNA evidence for variants both inside and outside the splice donor/acceptor ± 1,2 dinucleotides [8], based on alignment with a gene-specific or generic PVS1 decision tree [62]. Importantly, this splicing-focussed flowchart weights the evidence that a spliceogenic variant may be pathogenic, downweighting from the highest PVS1 (RNA) code if assay results indicate less severe functional consequences such as incomplete splicing impact, presence of rescue transcripts, or in-frame amino acid deletion outside clinically relevant domains. For RNA results showing multiple transcripts, the recommended approach is to assign a PVS1 strength to each transcript, group transcripts with the same strength, and then apply a conservative overall PVS1 level that reflects their relative contribution to total expression (Walker et al.,). With respect to application of construct-based evidence in variant curation and classification, current recommendations state that results for synonymous and intronic variants outside the splice region showing no impact relative to controls can be assigned a BP7_Strong (RNA) code [8]. However, there has been no ClinGen general recommendation on the maximum evidence weight towards pathogenicity applicable to construct-based data in the absence of calibration of an experimental system against clinical data for proven spliceogenic and non-spliceogenic variants. The decision tree for RNA analyses proposed by Buisine and colleagues [16] allowed a maximum weight of PVS1 for minigene RT-PCR data. The ClinGen ENIGMA BRCA1 and BRCA2 VCEP (Parsons et al., [50]) recommended that the overall evidence strength applicable to minigene RT-PCR data be downweighted and may not exceed PVS1_Strong (RNA) due to the artificial nature of minigene systems. Previous calibration analysis by O’Neill et al. [19] indicated that strong level of evidence was applicable for impact on splicing for *SCN1A* variants, as measured by their MPSA dataset. The results from the clinical calibration conducted in this study, which additionally assessed the level of predicted LOF transcripts induced by the variant, provide further justification to support application of strong level of evidence towards or against pathogenicity (PVS1_Strong (RNA), BP7_Strong (RNA) following current code recommendations) for RNA results from well-designed multi-exon minigene experiments.

We anticipate that the revisions to the ACMG/AMP guidelines, introduced in v4.0, will limit the maximum weight applicable for a variant that leads to transcript/s for which NMD is predicted to 6 points, but the overall consideration of how to assess if RNA results from constructs may be equivalent to those from patient material remain the same. Following current recommended processes, clinical calibration is not required to assign a PVS1 (RNA) code if the result is complete impact or no impact from assays using RNA from patient material, although it was suggested to consider adaptive weighting based on factors that may influence splicing assay results [8]. The value of clinical calibration is to set the aberration level threshold that is considered pathogenic (or benign) for variants that have partial impact on splicing and have been classified without use of RNA data. Construct-based studies can play a significant role here in providing allele-specific quantitative information to set the aberration level threshold to assign evidence towards or against pathogenicity for different gene-disease entities.

For MPSAs that do not return exact details on aberration type, assay-level calibration approaches are very important to derive an overall evidence weight for an individual study. As a first step, the LR can be calculated to assign evidence strength towards or against spliceogenicity for each splicing impact category. However for a true clinical calibration, several factors have to be considered: spliceogenic is not necessarily pathogenic (so some spliceogenic variants may justifiably be annotated as VUS or B/LB in ClinVar); non-spliceogenic is not necessarily benign (e.g. missense variants may justifiably be annotated as VUS or P/LP in ClinVar); ClinVar P/LP controls may have included splicing data in their classification introducing circularity (especially important for retrospective calibration); ClinVar LB controls may not be ideal negative clinical controls since variants can reach this classification with no clinical data (e.g. variant type and/or position (BP7) and bioinformatic prediction (BP4)). Having performed a study-wide calibration, assay-level and variant-level prediction information should preferably be used to inform further downweighting or upweighting of individual variant results from construct-based assays. For example, if there is a possibility of a multi-exon alternative event (based on literature, SAI-10k-calc or SpliceVault), then a single-exon construct design would not be able to capture this type of aberration, and a “no-impact” result should be discounted. Nevertheless, the same assay could provide valuable information to confirm predicted single exon skipping events, or predicted absence of an RNA impact. To allow increased clinical application of MPSA data consisting solely of assay scores such as delta PSI, we recommend the use of variant location information and bioinformatic predictions to assess likelihood of complete splicing and also the aberration type and relationship to pathogenesis. For example, given gene-specific knowledge, an in-frame deletion within a functional critical domain may be reasonably assigned evidence towards pathogenicity based on RNA findings, whereas an in-frame deletion outside a clinically important functional domain should not be considered evidence towards pathogenicity.

### General guidance to inform design and critique of construct-based assays for application in variant classification

Good experimental design, data quality control, and clinical validation are essential for the application of traditional minigene assay and MPSA results in clinical variant classification. General recommendations exist for the use of multiplexed functional data for clinical variant classification, but these are not specific for RNA findings, and cover a broad range of assays primarily measuring variant effects on protein function, cell growth, or cell viability [63]. In contrast, a recent publication from a multidisciplinary French network includes detailed recommendations for the experimental design of minigene splicing assays for diagnostic implementation [16]. Based on these previous publications, our critique and consideration of the performance of the MPSAs reviewed and our selected minigene dataset, and our observations about research reporting requirements to facilitate clinical application of RNA findings, we have compiled guidance for the application of traditional minigene assay or MPSA results in clinical variant classification. This guidance highlights modifications necessary to account for construct design limitations, different quantification methods, and the large-scale nature of MPSAs. We provide a broad overview of the most important considerations below, and more detailed information in Additional file 1: Table S13.

A clear understanding of the assay design, including knowledge of the types of splicing aberration that can (and cannot) be detected, is essential for accurately interpreting assay results. Sufficient intronic sequence flanking the exons is needed to ensure correct RNA splicing. Existing recommendations for traditional minigenes are that intronic sequence at both ends of the insert must be at least 150–200 bp [15, 16, 64], and this rationale holds true for MPSAs; O’Neill and colleagues [19] have demonstrated that it is possible to generate reliable MPSA results using constructs containing a single test exon with 125–250 bp of flanking intronic sequences in a MPSA. When designing new assays, we strongly recommend identification and formal quantification of transcripts. Since minigene assays do not fully preserve the physiological and genomic contexts, it is critical to assess the quality of the assay before it can be used for clinical variant classification.

Use of appropriate experimental controls is fundamental for establishing assay reliability, and comparison of minigene results with pre-existing splicing assay data is important to establish confidence in assay results. Both traditional minigene assays or MPSAs should include: (1) WT construct control to determine baseline splicing; in the context of residual alternative splicing events, normalising the level of spliced products from variant constructs to the WT value will be necessary for accurate interpretation of results; (2) No-template control to assess whether sample contamination has occurred; (3) Positive control variant(s) known to be spliceogenic, to assess the ability to detect splicing aberration relative to WT; and (4) Negative control variant(s) known to have no impact on splicing relative to WT (non-spliceogenic) to assess likelihood of false positives in a minigene context. While results using RNA from patient-derived material are generally set as the benchmark for detecting splicing aberration types since this is the clinical standard for validation, it should be recognised that every assay comes with its caveats, as demonstrated in this study.

We provide the following guidance for presenting the results of minigene assays, in particular MPSAs, to enhance their usability by clinicians and variant curators in diagnostic laboratories. Firstly, for variant description, genomic coordinates should be provided and defined according to a standard genome build [1], and the HGVS c. nomenclature must be reported based on the MANE Select transcript or the clinically relevant transcript [8]. Secondly, if the method is capable of characterising the spliced transcripts, we recommend providing the HGVS r. nomenclature based on the MANE Select transcript or the clinically relevant transcript. In addition, building on previous reports by the ENIGMA (Evidence-based Network for the Interpretation of Germline Mutant Alleles) consortium, we advise using the shorthand nomenclature adapted and extended from Colombo et al. [65] to help simplify transcript annotation and facilitate variant curation. This additional aberrant transcript nomenclature, described in detail in Additional file 1: Table S14, enables easy identification of affected exons or introns and the sizes of deleted or retained sequences. Since this nomenclature style does not indicate the exact change in the transcript nucleotide sequence, it must always be accompanied by the HGVS description. We also recommend that transcripts be annotated to indicate whether they result in LOF or not. Thirdly, if the method can formally quantify transcripts, we recommend recording the percentages of variant-induced full-length and aberrant transcripts. For MPSAs, thresholds enabling categorical annotations of splicing impact, such as “abnormal splicing”, “indeterminate”, or “normal splicing”, must be provided to facilitate the interpretation of assay scores. Fourthly, data and experimental details must be deposited on publicly accessible databases and repositories. Further information on disclosure of datasets and statistical methods for MPSAs is detailed in Gelman et al. [63]. We anticipate this guidance may aid the design of new minigene splicing assays intended to have clinical application, and the quality assessment of data from existing minigene splicing assays.

## Conclusions

There is no formal guidance on optimal assay design and validation of construct-based RNA results for application within the ACMG/AMP variant interpretation framework. We highlight that it is essential to evaluate the design limitations and performance of broad MPSA screens before considering their use in clinical variant classification, either directly or indirectly. We have shown that only one of seven MPSA datasets evaluated demonstrated performance suitable for clinical application of the RNA evidence in variant classification.

These findings also raise concerns about use of MPSA datasets for benchmarking of new prediction tools. By extension, our findings have important implications for use of public collated splicing datasets in research or clinical studies. For example, the SpliceVarDB repository of splicing assay data was created to improve access to variant-specific splicing information for curators and researchers [66], and includes splicing assay data from multiple MPSA studies. Our findings suggest the value for quality control assessment of any studies included in such a repository.

We have also shown that evaluation and analysis of well-designed multi-exon minigene assays reliably recapitulate patient splicing outcomes, and when clinically calibrated, can provide strong evidence towards or against pathogenicity using the ACMG/AMP variant classification framework. Analysis of this quantitative construct-based data highlighted potential for a more nuanced interpretation of splicing impact prediction, by demonstrating that SpliceAI score bins predict the level of splicing impact, and SAI-10k-calc accurately predicts variant-induced aberration types and their functional consequences.

## Supplementary Information

Additional File 1: Table S1 Description of published traditional minigene RT-PCR and MPSA datasets selected for inclusion in analysis; Table S2 Detailed summary of MPSA designs and characteristics; Table S3 Traditional minigene RT-PCR splicing assay results and bioinformatic predictions; Table S4 Variants tested by RT-PCR minigene assay and reported in ClinVar with clinical classifications; Table S5 Manual review of ClinVar submissions for variants outside the splice donor/acceptor ±1,2 dinucleotide positions; Table S6 Concordance of SpliceAI max delta score thresholds defined in Walker et al., 2023 and observed splicing by MPSAs; Table S7 Traditional minigene structures, number of variants tested, and percentage of canonical transcript produced by each wild type construct; Table S8 Distribution of variants outside the splice donor/acceptor ±1,2 dinucleotide positions with additional stratification of SpliceAI max delta score bins; Table S9 Proportion of variants (including the splice donor/acceptor ±1,2 dinucleotide positions) across different ranges of SpliceAI max delta scores; Table S10 Concordance of SAI-10k-calc transcript prediction with traditional minigene assay results; Table S11 Concordance of traditional minigene and patient RNA findings; Table S12 BRCA2 variants showing no aberration in patient RNA assays; Table S13 Considerations for design and critique of minigene and massively parallel splicing assay reliability and performance; Table S14 Shorthand nomenclature for variant-induced transcripts.

Additional File 2: Figure S1. Decision flowchart for SNVs flagged by SAI-10k-calc for manual inspection of SpliceAI scores using SpliceAI-visual. Figure S2. Summary of MPSA performance of variant relative to distance to splice donor/acceptor ±1,2 dinucleotides.

## Data Availability

No datasets were generated or analysed during the current study.

## Abbreviations

ACMG: American College of Medical Genetics and Genomics
AMP: Association for Molecular Pathology
B/LB: Benign / Likely Benign
ClinVar: Clinical Variant database
gnomAD: Genome Aggregation Database
LOF: Loss of Function
LR: Likelihood Ratio
MANE: Matched Annotation from NCBI and EMBL-EBI
MaPSy: Massively Parallel Splicing (Yeast-based) Assay
MFASS: Multiplexed Functional Assay of Splicing using Sort-seq
MPSA: Massively Parallel Splicing Assay
NMD: Nonsense-Mediated Decay
P/LP: Pathogenic / Likely Pathogenic
ParSE-Seq: Parallel Splice Effect Sequencing
PSI: Percent Spliced In
PTC: Premature Termination Codon
RNA-seq: RNA sequencing
ROC: Receiver Operating Characteristic
RT-PCR: Reverse Transcription Polymerase Chain Reaction
VCEP: Variant Curation Expert Panel
VEP: Variant Effect Predictor
Vex-seq: Variant Exon Sequencing
VUS: Variant of Uncertain Significance
WT: Wild Type
