# Supplementary material for: Evidence-based recommendations for application of construct-based splicing data in clinical variant classification
Source: Genome Med. 2026 Aug 13;18:176. doi: 10.1186/s13073-026-01746-3 (PMC13753216; doi:10.1186/s13073-026-01746-3)
Supplement: Supplementary file 1 [file 13073_2026_1746_MOESM1_ESM.docx]

**Additional file 2**

**Figure S1.** Decision flowchart for SNVs flagged by SAI-10k-calc for manual inspection of SpliceAI scores using SpliceAI-visual.

**Figure S2.** Summary of MPSA performance of variant relative to distance to splice donor/acceptor ±1,2 dinucleotides

**Fig. S1** Decision flowchart for SNVs flagged by SAI-10k-calc for manual inspection of SpliceAI scores using SpliceAI-visual. The SAI-10k-calc algorithm for predicting exon skipping and intron retention requires the loss of both native donor and acceptor splice sites. The flag “lost site/s do not match native site” means that at least one of the SpliceAI-predicted losses affects a cryptic splice site. While SpliceAI provides the location of acceptor and donor loss (and gain) with the highest delta scores only, SpliceAI-visual displays all the SpliceAI-predicted splice site loss/gain across the transcript and can reveal a predicted loss of a native donor-acceptor pair that might be missed by SpliceAI only. Prediction of exon skipping or intron retention requires at least one of the lost native sites to have a SpliceAI score ≥0.2.

**Fig. S2** Summary of MPSA performance of variant relative to distance to splice donor/acceptor ±1,2 dinucleotides**.** Each MPSA is summarised in a single panel with three rows (I-III). The exonic regions are depicted as boxes with the representative study name and the intronic regions as a black line. The three studies that used the MaPSy assay(1-3) are combined as one panel. Row I) A histogram of the number of variants (magenta) found at varying distances bins from the exon boundaries. Row II) Boxplots of the respective MPSA scores and study-reported assay thresholds shown as a red dashed line. For Patel et al., the MPSA results are presented as the proportion of reported “splice-affecting” (orange) and “benign” (grey) variants. Row III) Boxplots of SpliceAI max delta scores for variants in each bin, with the threshold for predicted spliceogenicity (0.2) as a red dashed line. SAI, spliceAI; PSI, percent spliced in.

**REFERENCES**

1.	Rhine CL, Neil C, Wang J, Maguire S, Buerer L, Salomon M, et al. Massively parallel reporter assays discover de novo exonic splicing mutants in paralogs of Autism genes. PLoS Genet. 2022;18(1):e1009884.

2.	Rong S, Neil CR, Welch A, Duan C, Maguire S, Meremikwu IC, et al. Large-scale functional screen identifies genetic variants with splicing effects in modern and archaic humans. Proc Natl Acad Sci U S A. 2023;120(21):e2218308120.

3.	Soemedi R, Cygan KJ, Rhine CL, Wang J, Bulacan C, Yang J, et al. Pathogenic variants that alter protein code often disrupt splicing. Nat Genet. 2017;49(6):848-55.
